# Spiky Durian-Shaped Au@Ag Nanoparticles in PEDOT:PSS for Improved Efficiency of Organic Solar Cells

**DOI:** 10.3390/ma14195591

**Published:** 2021-09-26

**Authors:** Muheeb Ahmad Alkhalayfeh, Azlan Abdul Aziz, Mohd Zamir Pakhuruddin, Khadijah Mohammedsaleh M. Katubi

**Affiliations:** 1 School of Physics, Universiti Sains Malaysia, USM, Pulau Pinang 11800, Malaysia; zamir@usm.my; 2 Chemistry Department, College of Science, Princess Nourah Bint Abdulrahman University, Riyadh 11671, Saudi Arabia; kmkatubi@pnu.edu.sa

**Keywords:** organic solar cell, concentration ratio NPs, LSPR, plasmonic effect, Au@Ag NPs

## Abstract

The localized surface plasmon resonance (LSPR) effects of nanoparticles (NPs) are effective for enhancing the power conversion efficiency (PCE) of organic solar cells (OSCs). In this study, spiky durian-shaped Au@Ag core-shell NPs were synthesized and embedded in the hole transport layer (HTL) (poly(3,4-ethylenedioxythiophene): poly(styrenesulfonate) (PEDOT:PSS)) of PTB7:PC_71_BM bulk-heterojunction OSCs. Different volume ratios of PEDOT:PSS-to-Au@Ag NPs (8%, 10%, 12%, 14%, and 16%) were prepared to optimize synthesis conditions for increased efficiency. The size properties and surface morphology of the NPs and HTL were analyzed using field emission scanning electron microscopy (FESEM), transmission electron microscopy (TEM), and atomic force microscopy (AFM). UV–Vis spectroscopy and current density–voltage (J-V) analysis were used to investigate the electrical performance of the fabricated OSCs. From the results, we observed that the OSC with a volume ratio of 14% (PEDOT:PSS–to–Au@Ag NPs) performed better than others, where the PCE was improved from 2.50% to 4.15%, which is a 66% increase compared to the device without NPs.

## 1. Introduction

The quest for novel and eco-friendly energy solutions such as solar photovoltaics has increased rapidly as a viable alternative to fossil fuels, which have been contributing to abnormal climate conditions, principally the current global warming situation. Therefore, the market for renewable energy sources is expanding. In particular, organic solar cells (OSCs) recently received widespread interest owing to the low cost of synthesis and fabrication, facile processing procedures, tunable optical characteristics, and flexibility. However, the OSCs are constrained by low power conversion efficiency (PCE) and open-circuit voltage (V_oc_) due to their inadequate light absorption, low charges diffusion length, and high recombination rate leading to exciton quenching in the active layer [1,2,3]. As such, OSCs have been modified or functionalized to boost the capacity of the thin active layer to absorb light by means of periodic grating structures in electrodes, controlling the morphology of the layers, reconfiguring the optical device structure for light distribution, and incorporating nanoparticles (NPs). Among these strategies, NPs have gained more interest as an efficient approach for trapping light in the photoactive layer and improving the dissociation of excitons because of their near-field coupling effect [4,5,6,7,8,9,10,11,12,13].

The incorporation of NPs into the buffer layer (i.e., poly(3,4-ethylenedioxythiophene):poly(styrenesulfonate) (PEDOT:PSS) takes advantage of the electrical hole collection enhancement, far-field scattering effect, and localized surface plasmon resonance (LSPR) by initiating chemical and morphological changes in the buffer layer. PEDOT:PSS was utilized as a hole transport layer (HTL) because of its high transparency in the visible range, electrical conductivity, lasting stability, simple processing, and the usage of ecofriendly solvent [13,14,15]. Moreover, PEDOT:PSS was used as transparent composite electrodes (TCEs) by doping copper nanowires (CuNWs), which demonstrated a high transmittance of about 90% at a wavelength of 460 nm and the potential for application in flexible solar cells and wearable electronics [16]. Exciton quenching can also be avoided by identifying the sites of NPs at the indium tin oxide (ITO) or at the buffer layer, or between the active and buffer layers. The effect of incorporating NPs of gold (Au) and silver (Ag) on the performance of OSCs was investigated because of the relatively high scattering strength, chemical stability, and remarkable LSPR optical properties. Moreover, Au NPs are characterized by high conductivity and biocompatibility, and they can improve the sensitivity and stability of devices and the facilitation of accepted electron transfer towards the electrode [17,18]. Several metal nanostructures have been functionalized to significantly augment the enhancement of light absorption, such as the composite NPs with different metals [19,20,21], Au nonspherical [22] and nanorods [23], Au nanostars [24], Ag with multiple-shaped [25], truncated octahedral [26], decahedral/icosahedron Ag NPs [27], mixing of the Au and Ag NPs [28], Au NPs with graphene shell (Au NPs@Gr) [29], rough-surface Au@Ag core-shell NPs (RSAu@Ag NPs) [30], Au@Ag nanocube (NC) [31], and durian-shaped Au@Ag NPs (numerous pointed spikes) [32]. The PCE of OSCs embedded with durian-shaped Au@Ag NPs is more enhanced than that of OSCs fabricated with composite Au@Ag NPs, due to the integrative impact of Au NPs’ strong spectral response and Ag NPs’ high scattering power at the long-wavelength range. In addition, the tiny radii of Au@Ag NPs’ spike endings considerably improve the EM field at their sites, which significantly augments the PCE of OSCs [32]. However, the performance of OSCs embedded with NPs strongly depends on the density of NPs in the HTL. The further increment of NPs density in HTL such as PEDOT:PSS would be harmful to the morphology of the active layer and would also cause a substantial loss of reflection of the incident light.

In this study, core/shell structure of spiky durian-shaped Au@Ag NPs is embedded into PEDOT:PSS to achieve optimized cell performance through the appropriate concentration of Au@Ag NPs in PEDOT:PSS. These OSCs are dependent on the active layer of PTB7:PC_71_BM thin film that donates and accepts charged species, respectively. The size properties and surface morphology of Au@Ag NPs and the electrical performance of the OSCs embedded with Au@Ag NPs under various volume ratios of PEDOT:PSS-to-Au@Ag NPs are examined using FESEM, TEM, UV-Vis spectrometer, and AFM. The impact of Au@Ag NPs with varying doping ratios on the PCE is investigated to attain the optimal settings for improved PCE. As a result, we obtained 66% enhancement of PCE and achieved 56% improvement in short-circuit photocurrent density (J_sc_) compared to the reference OSCs.

## 2. Experimental

### 2.1. Materials

A variety of materials were utilized to synthesize the Au@Ag NPs and fabricate the OSC. Gold chloride trihydrate (HAuCl_4_.3H_2_O), polyvinylpyrrolidone with M mass 10–4 g/mol (PVP), silver nitrate (AgNO_3_), ascorbic acid (AA, 99.7%), and ITO-coated glass substrates with a resistance of 12–22 Ω/sq were purchased from Sigma-Aldrich (St. Louis, MO, USA). Isopropyl alcohol (IPA), ethanol, and acetone were utilized to wash the ITO glass, while hydrochloric acid (HCl) and zinc metal (Zn) were used to etch out the ITO electrode from certain parts of the glass slide. The buffer layer was a composite of PEDOT:PSS. The active layer was a compound of [4,8bis[(2ethylhexyl)oxy]-benzo[1,2-b:4,5-b’]dithiophene-2,6-diyl][3fluoro2 [(2ethylhexyl)-carbonyl]-thieno-[3,4-b] thiophenediyl (PTB7), and [6,6]-Phenyl (C_71_) butyric acid methyl ester, and a mixture of isomers (P C_71_BM) were dissolved in 1,8-diiodooctane and chlorobenzene (CB). All these materials were procured from Sigma-Aldrich. The experiments utilized ultra-pure de-ionized (DI) water.

### 2.2. Synthesis of Au@Ag NPs

Au@Ag NPs were prepared from an aqueous composite solution of Ag and Au. The precursor solutions include 20 µL of 10 mM AgNO_3_ and 200 µL of 10 mM HAuCl_4_, combined with 10 mL de-ionized water. Following the incorporation of the mixed, freshly made AA (100 mM) with molar ratios of 40 µL, the obtained solution was stirred for 20 s. The change in color of the mixed solution to blue implies the development of durian-shaped Au@Ag NPs. Afterward, 2 mg of PVP dissolved in 80 µL of water was included to stabilize the solution.

### 2.3. Fabrication of OSC Device

**Figure 1:**
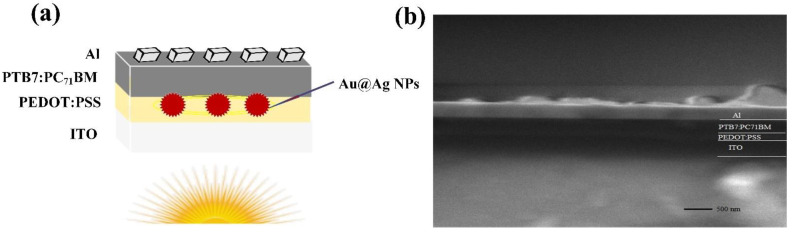
(**a**) Schematic of the OSC and (**b**) FESEM cross-section of the OSC.

The OSC device was fabricated as a configuration of ITO/PEDOT:PSS+Au@Ag NPs/active layer/Al. The respective devices were fabricated in compliance with the following procedures: (1) ITO-glass substrate: Zn and HCl were utilized to etch the ITO electrode from certain parts of the glass slide to electrically isolate the positive and negative electrodes. The substrate was subsequently washed with detergent, ultrasonicated in acetone, ethanol, IPA, and DI water for 20 min to eliminate organic residues, and dried in an oven at 110 °C for 10 min. (2) Buffer layer: PEDOT:PSS was employed as an HTL due to its high transparency in the visible part of the spectrum, non-toxic and long-term stability [33]. These exceptional properties make it ideal as an interfacial/electrode material in the fabrication of OSCs [22,34,35,36]. The PEDOT:PSS solution was placed in ultra-sonicate for 30 min and filtered through a 0.45 µm nylon filter to form a high-quality layer. In this regard, the volume ratios of PEDOT:PSS-to-Au@Ag NPs solution were set at 8%, 10%, 12%, 14%, and 16% (sample S2–S6). The reference cell (S1) did not contain NPs. The PEDOT:PSS, in the presence and absence of Au@Ag NPs, was evenly distributed on the ITO glass, followed by rotation at 3500 rpm for 30 s using a spin-coater to obtain a thickness of about 40 nm. (3) Active layer: a fixed-ratio of PTB7 and PC_71_BM 1:1.5 based on PTB7 concentration was blended overnight in a glove box, which was then filtered through a 0.2 µm PTFE filter, and deposited on PEDOT:PSS by spin-coating at 1100 rpm for 30 s to obtain an active layer thickness of approximately 100 nm. (4) Electrode: 100 nm thick Al was evaporated at a pressure level of 4 × 10^−5^ mbar due to its techno-friendliness and low-cost. The active area of the device was about 0.25 cm^2^, which comprised an overlap area of the Al and ITO layers. Figure 1a presents the schematic of an OSC device embedded with plasmonic Au@Ag NPs, while Figure 1b shows the FESEM cross-section image of the OSC.

### 2.4. Sample Description and Measurements

The size and morphology of the durian-shaped Au@Ag NPs were characterized using FESEM and TEM. Before the TEM analysis, aliquots of the Au@Ag NPs were air-dried at room temperature after being installed on a carbon-coated copper grid. UV-Vis spectrophotometer was utilized to obtain the absorption spectra of ITO/PEDOT:PSS multilayer film in the presence and absence of Au@Ag NPs/PBT7: PC_71_BM. Furthermore, the surface morphology of the PEDOT:PSS film, in the presence and absence of durian-shaped Au@Ag NPs, was characterized utilizing AFM (Bruker Dimension Edge microscope). After the fabrication, current density–voltage (J-V curve) measurements were carried out under ambient conditions using Keithley 2400 source measurement unit and solar simulator at AM 1.5 G.

## 3. Results and Discussion

### 3.1. Morphology Study

**Figure 2:**
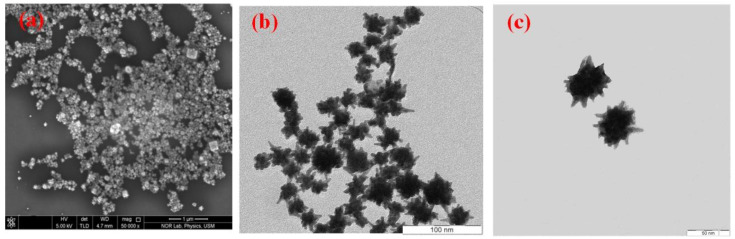
Spiky durian-shaped Au@Ag NPs (**a**) FESEM image with 1 µm scale, (**b**) TEM image with 100 nm scale, and (**c**) higher resolution TEM image with 50 nm scale.

**Figure 3:**
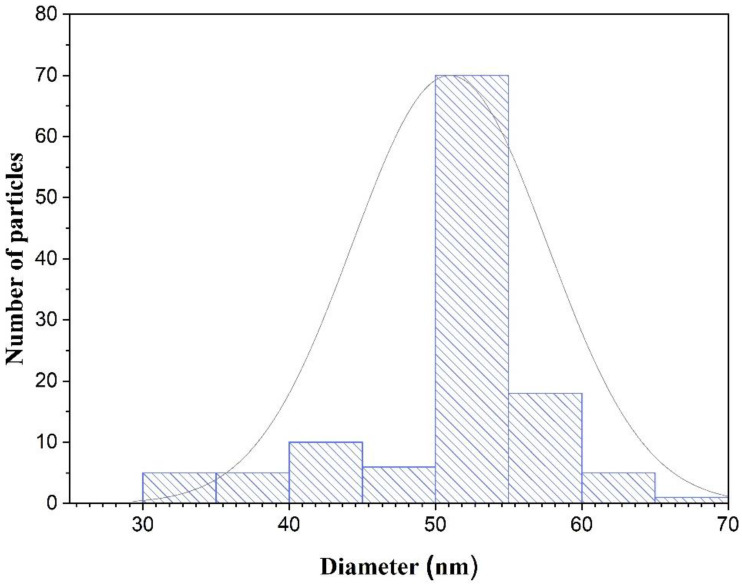
Size distributions of the synthesized Au@Ag NPs.

As previously discussed, the durian-shaped Au@Ag NPs were synthesized via reduction of the combined HAuCl_4_ and AgNO_3_ solution using ascorbic acid. The ratio between the NPs and precursor HAuCl_4_/AgNO_3_ solution was modified to achieve the durian shape. Reactions occurred in the precursor solution immediately after the introduction of L-ascorbic acid at room temperature. Furthermore, size control in the synthesis of Au NPs has been reported in several studies [37,38,39]. The ascorbic acid selectively reduces the Au ions in the HAuCl_4_/AgNO_3_ mixed solution to form Au NPs due to their higher reducing potential than the Ag ions. Afterward, the residual or non-reactive phase of Ag ions is deposited on the surface of Au NPs to form sharp spikes [40]. Figure 2a presents the FESEM image of the Au@Ag NPs. TEM images of the Au@Ag core-shell nanostructure are presented in Figure 2b. Notably, the majority of the particle dispersion is homogenous without agglomeration. The small dark particles discernible in Figure 2b,c represent the Au NPs. The well-developed durian (spikes) shaped Au@Ag NPs observed from the high-resolution TEM images (Figure 2c) were obtained for precursor concentrations of 200, 20, and 40 µL for HAuCl_4_, AgNO_3_, and AA, respectively. The size distribution of the synthesized NPs is presented in the histogram shown in Figure 3, where the dominant diameter range is approximately between 50 and 55 nm.

**Figure 4:**
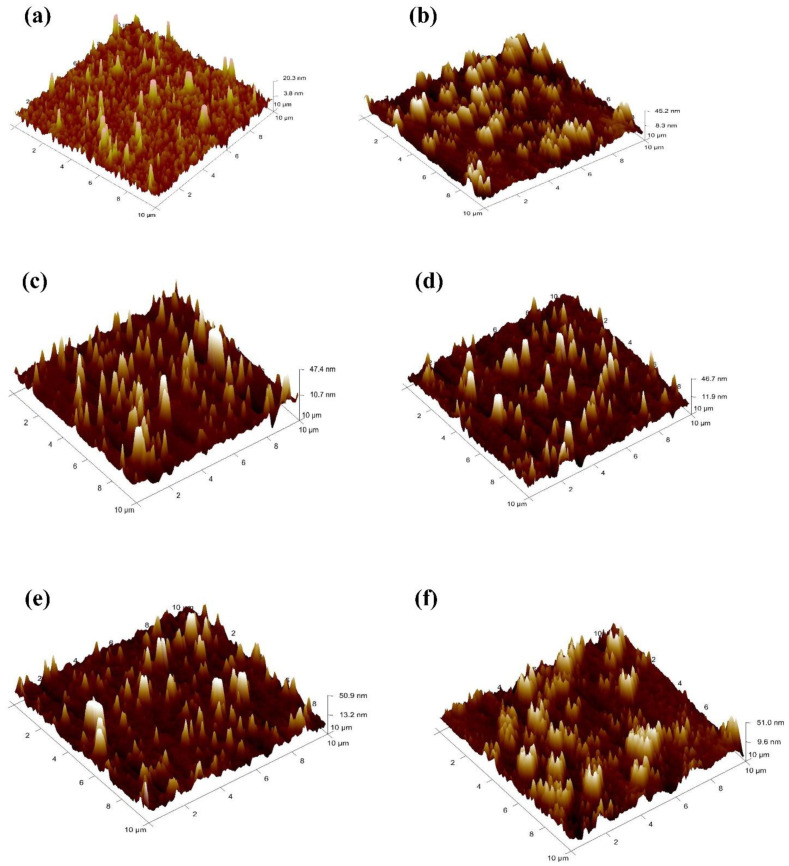
The AFM topographical images of the buffer layer (**a**) PEDOT:PSS only (without NPs, S1), (**b**) Au@Ag-8% (S2), (**c**) Au@Ag-10% (S3), (**d**) Au@Ag-12% (S4), (**e**) Au@Ag-14% (S5), (**f**) Au@Ag-16% (S6).

Figure 4 represents the 3D atomic force microscopy (AFM) images of PEDOT: PSS, before and after embedding Au@Ag NPs. Although the root mean square (RMS) of surface roughness of PEDOT:PSS deposited on the ITO layer without NPs is 1.1 nm, the RMS increased to 8.1, 10.3, 9.1, 10.7, and 11.6 nm after embedding the PEDOT:PSS with Au@Ag NPs at volume ratios of 8%, 10%, 12%, 14%, and 16% respectively. The increase in the embossed RMS roughness is possibly due to the changes in the size of Au@Ag NPs; the higher volume ratio of Au@Ag NPs embedded in PEDOT:PSS, and higher amount of sharp spikes that penetrates the PEDOT:PSS film [27,29,41,42]. The spikes of Au@Ag NPs could pierce the active layer, leading to higher amounts of generated excitons and the development of SPR at the interface between PC_71_BM and PTB7, which enhances the dissociation of excitons. In contrast, the further increment in concentrations of Au@Ag NPs (i.e., volume ratio of Au@Ag NPs into the buffer layer) would be detrimental to the morphology of the active layer and generates a considerable device reflection loss as observed in S6.

### 3.2. UV-Vis Spectroscopy

**Figure 5:**
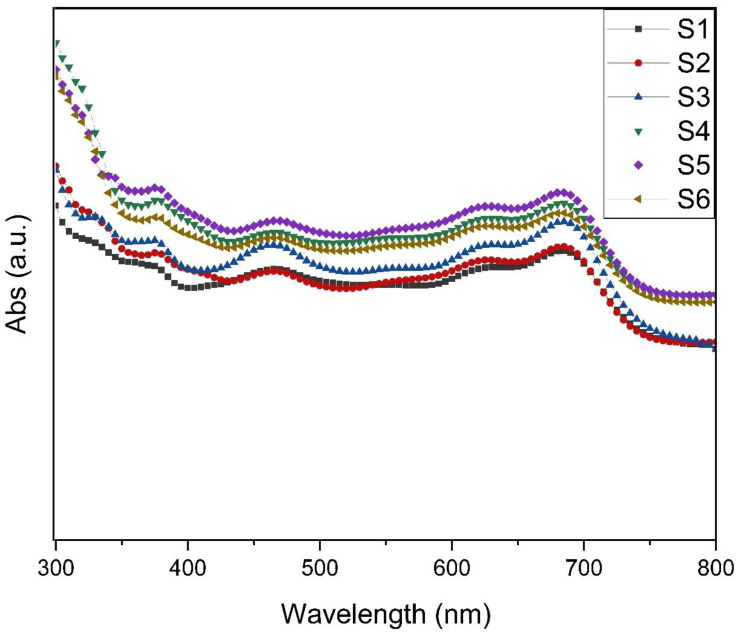
UV-Vis absorption spectra of the OSCs with and without Au@Ag NPs embedded into PEDOT:PSS at different ratios.

For direct investigation of the effects of LSPR and to further illustrate enhancements in the absorption and harnessing of the incident light in the active layer with/without NPs, a comparison was made between the UV-Vis absorption spectra of the layers (ITO/PEDOT:PSS/PTB7:PC_71_BM), in the presence and absence of Au@Ag NPs in the PEDOT: PSS, as seen in Figure 5. The substantial LSPR field contiguous to the NPs improves the broad wavelength range. The improved absorption of light in the photoactive layer leads to increased generation of excitons (e–h pairs). Compared to the reference cell, the films embedded with Au@Ag NPs demonstrate higher absorptions in the range of 300–800 nm, which is possibly due to enhancement in EM resulting from the SPR phenomenon of the NPs. In addition, further broad absorption possibly increases the electric field intensity in PTB7:PC_71_BM, because of the small radii at the ends of the spikes that penetrates the active layer, and the elongated optical path length initiated by SPR activated scattering effects of the NPs embedded into the buffer layer [24,30,43,44]. The most significant improvement in light absorption due to incorporating the Au@Ag NPs is observed at a volumetric ratio of 14% (S5). However, increasing the number of NPs embedded in the buffer layer leads to a significant device reflection loss, as observed in S6. Finally, the number of NPs serves a vital function in light absorption improvement in the active layer and may affect electrical properties.

### 3.3. The Current Density–Voltage of OSCs with/without Embedding Au@Ag NPs into the Buffer Layer

**Figure 6:**
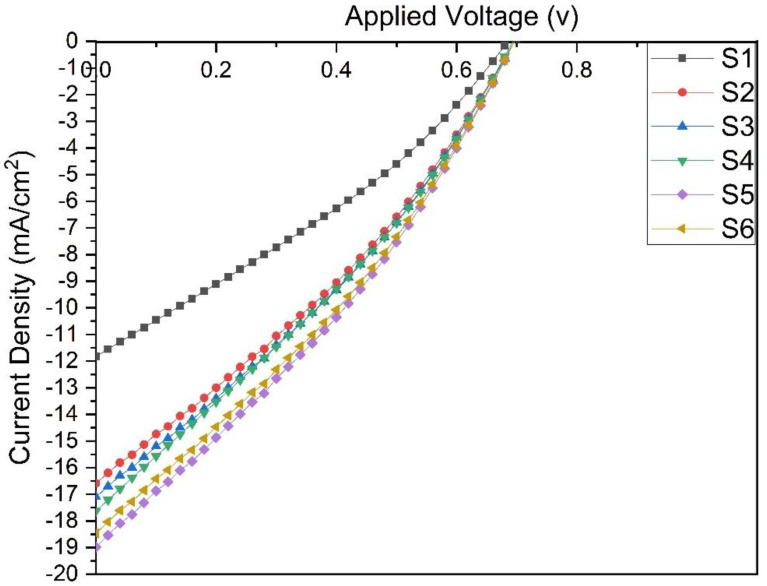
J–V curves of OSCs with and without Au@Ag-(8%, 10%, 12%, 14%, and 16%) NPs.

Enhanced photovoltaic efficiency can be attributed to the fast generation of excitons at PTB7 and the plasmonic effect of the embedded NPs as a donor layer and decomposition at the interfacial layer separating the donor-acceptor (PC_71_BM). The light is more amplified into the donor layer to be absorbed in PTB7 in this phenomenon and according to PEDOT:PSS with embedded plasmonic NPs, the built-in field at the interfacial layer is enhanced. The SPR is referred to as the electron oscillation of NPs induced by the association between the incident light and the NP’s surface. The resonance state arises as the frequency of the incident photons becomes equivalent to the normal frequency of the metallic electrons against the restoring force of the positive nuclei. To achieve better performance of the OSCs by incorporating NPs, it is crucial to choose a suitable volume ratio of the Au@Ag NPs embedded into the PEDOT:PSS. Electricity generated by a photovoltaic cell is reliant on the device’s J_sc_ and V_oc_ values, as well as the fill factor (FF) at a particular incident power (P_in_), according to the equation of PCE ($\mathrm{PCE}=J_{\mathrm{sc}}\cdot V_{\mathrm{oc}}\cdot FF/P_{in}).$ J_sc_ and V_oc_ are obtained using a solar simulator (AM1.5G) and FF using the formula $(FF=(J_{\max}\cdot V_{\max})/\left(J_{sc}\cdot V_{oc}\right)$). The maximum current (J_max_) and voltage (V_max_) are obtained at the maximum output power point (P_max_). J–V properties of ITO/PEDOT:PSS embedded with different volume ratios of Au@Ag NPs/PTB7: BC_71_BM/Al solar cells are presented in Figure 6, while their PV parameters are explained in Table 1.

The PCE of the reference device, i.e., PEDOT:PSS without Au@Ag NPs, is 2.5%. The embedding of the Au@Ag NPs in PEDOT:PSS to form HTL increases the PCE to 3.60% for Au@Ag NPs-8% (S2), 3.73% for Au@Ag NPs-10% (S3), 3.84% for Au@Ag NPs-12% (S4), 4.15% for Au@Ag NPs-14% (S5), and 4.02% for Au@Ag NPs-16% (S6). After introducing the Au@Ag NPs into the PEDOT:PSS film, the FF and Voc values differ from those of the reference cell (S1). Nonetheless, a significant enhancement in relative J_sc_ is observed, which explains the improved PCE of the hybrid HTL films. When compared to the reference device, the higher J_sc_ of the hybrid HTL films can be attributed to the corresponding enhancement of the light absorption by the active layer due to the presence of Au@Ag NPs, which scatter the incident light. Moreover, the improvement in the J_sc_ from 11.82 to 18.46 mA/cm^2^ indicates the enhanced generation of excitons and their dissociation to e-h pairs by the plasmonic effects. Generally, the shape of Au@Ag NPs plays a vital role, given that it has a direct effect on both SPR and scattering of the incident light.

**Figure 7:**
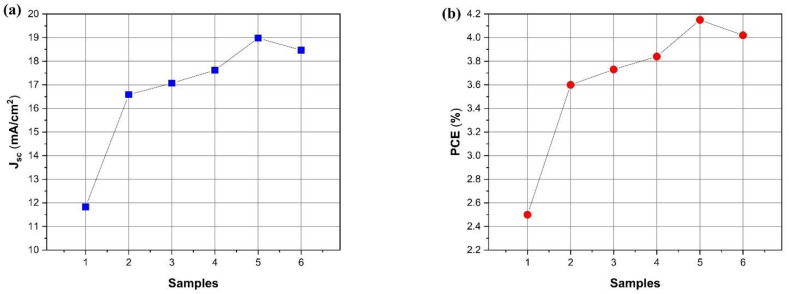
(**a**) Jsc and (**b**) PCE of the devices with and without NPs (S1–S6).

Adding the Au@Ag NPs with numerous pointed spikes into PEDOT:PSS improves the PCE of OSCs, probably due to the tiny radii of the spikes’ tips, leading to significant improvement in the EM field [32]. Furthermore, the embedding of Au@Ag NPs into the buffer layer leads to a modification of the work function [42]. Accordingly, the PCEs of the OSCs increase (relative) by 44%, 49%, 53%, 66%, and 60% when the PEDOT:PSS is embedded with Au@Ag NPs with volume ratios 8%, 10%, 12%, 14%, and 16%, respectively. The increments in the J_sc_ and PCE of the OSCs (Figure 7a,b) are evident after embedding the PEDOT:PSS layer with different volume ratios of plasmonic Au@Ag NPs, when compared to the reference cell. This can be attributed to an increase in the effect of the LSPR due to the volume ratio of Au@Ag NPs to PEDOT:PSS, which improves the light absorption in the active layer (as demonstrated in Figure 5).

## 4. Conclusions

This study demonstrates the enhancement effect of embedding the composite of Au@Ag NPs (which consists of spiky, durian-shaped plasmonic cores) in PEDOT:PSS on the PCE of OSCs. The enhancement is related to increased light-trapping, attributable to LSPR produced by the Au@Ag NPs and increased device conductivity. In addition, the volume ratio of Au@Ag NPs into PEDOT:PSS serves a vital function in the enhancement of the PCE. As indicated by the results, the optimized OSC shows a peak PCE of 4.15%, which is 66% higher than the reference sample. The improvement is mainly coming from the J*_sc_*, which increases from 11.82 to 18.46 mA/cm^2^. This work offers a technique focused on the core-shell configuration of Au@Ag NPs to achieve high-efficiency plasmonic-enhanced OSCs, and this technique could be applicable to other types of solar cells.

## Figures and Tables

**Table 1:** Electrical parameters of the S1-S6.

Samples	J*_sc_* (mA/cm^2^)	V*_oc_* (mV)	J*_max_* (mA/cm^2^)	V*_max_*(mV)	FF (%)	PCE(%)
S1	11.82	685.8	6.27	400	30.9	2.50
S2	16.58	696.4	9.00	400	31.2	3.60
S3	17.07	697.5	9.32	400	31.3	3.73
S4	17.62	698.0	9.62	400	31.3	3.84
S5	18.98	699.1	10.41	400	31.4	4.15
S6	18.46	698.6	10.06	400	31.2	4.02

## Data Availability

Not applicable.
